# Hypertrophic Cardiomyopathy Mutation R723G in *MYH7* Enhances Its mRNA‐Stability

**DOI:** 10.1111/apha.70233

**Published:** 2026-05-15

**Authors:** Kathrin Kowalski, Luisa Schwarze, Theresia Kraft, Judith Montag

**Affiliations:** ^1^ Molecular and Cell Physiology Hannover Medical School Hannover Germany; ^2^ Department of Human Medicine Medical School Berlin Berlin Germany

Allelic imbalance describes the unequal expression of a gene's two alleles. Whereas it has no effect under normal circumstances, it can aggravate disease course in patients with heterozygous mutations. Hypertrophic cardiomyopathy (HCM), the most common inherited cardiac disorder, is mostly caused by heterozygous mutations in different sarcomeric proteins. One of the most commonly affected proteins is the β‐myosin heavy chain (β‐MyHC), encoded by *MYH7*. In several HCM patients, unequal fractions of mutant mRNA and protein have been detected and higher fractions of mutant protein have been associated with a more severe disease course [1, 2]. However, it is unknown which mechanisms cause the unequal allelic ratios in the HCM patients with missense mutations.

For HCM‐mutation R723G we have shown previously that patients express on average 67% of mutant and 33% of wildtype mRNA and protein [2]. This increased amount of mutant mRNA was also maintained in patient‐derived hiPSC‐CMs (Figure 1A, *n* = 6 hiPSC‐CM differentiations), in line with previous findings [3]. We have further shown that mutation R723G can alter mRNA secondary structure [4]. We hypothesize that this could alter mRNA turnover and thereby explain the increased allelic fraction of R723G‐mRNA. In this case, either production of R723G‐mRNA or its stability could be affected. Thus, we compared transcription and degradation of R723G and WT‐mRNA to identify potential mechanisms.

**FIGURE 1:**
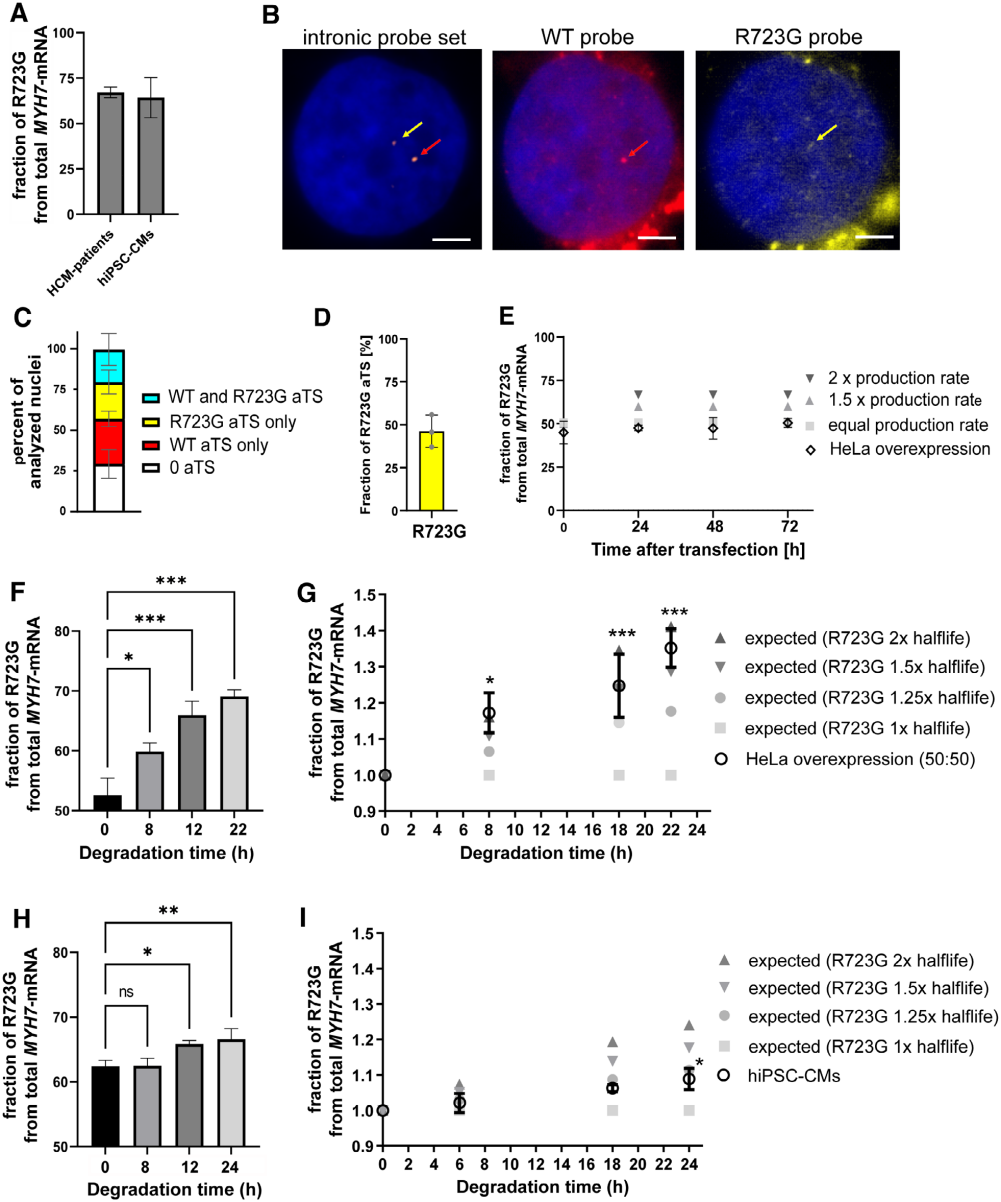
Transcription and degradation of *MYH7* wildtype and R723G‐alleles. (A) Fraction of WT per R723G‐mRNA from *n* = 7 different HCM patients and hiPSC‐CMs from *n* = 6 differentiations were analyzed by RT‐PCR and allele specific restriction analysis [2]. (B) Exemplary RNA‐FISH analysis with allele specific probes in a hiPSC‐CM nucleus stained by DAPI. hiPSC‐CMs were hybridized with an intronic probe set (left panel) to visualize *MYH7*‐pre‐mRNA [3] and with a WT‐specific probe (middle panel) and a R723G‐specific probe (right panel). Allele specific probes were 28 bp long; 10 bp (WT: 5′‐ATGCGATACC‐3′ and R723G: 5′‐ATGCCATACC‐3′, respectively) were single stranded and 18 bp (5´‐TGAGGAGGGAAGTGTCCA‐3′) were designed with a mask oligo (5′‐TGGACACTTCCCTCCTCA‐3′). Co‐localization of the intronic probe and either WT‐ or R723G‐probes indicated an active transcription site. Scale bar represents 5 μm. (C) Quantification of allele specific transcription analyzed by RNA‐FISH. A total of 121 nuclei from 3 differentiations were analyzed. They either showed no active transcription (0 aTS, white bar), transcription of only the WT‐allele (red bar), transcription of only the R723G‐allele (yellow bar), or transcription of both alleles (cyan bar). (D) Fraction of nuclei that express R723G‐allele derived from RNA‐FISH analysis (*n* = 3 differentiations). (E–G) HeLa cells were transfected with equal amount of WT and R723G‐encoding expression vector. (E) The transcribed fraction of WT per R723G‐mRNA was analyzed by RT‐PCR and allele‐specific restriction analysis after 24, 48 and 72 h and compared to expected ratios for different increased production rates of R723G‐mRNA. (F) Transcription was blocked using Actinomycin D and WT per R723G‐ratios were analyzed after 8, 18 and 22 h. (G) Fold change of R723G‐mRNA was calculated for different expected half‐life (WT: 8 h) and compared to the experimentally determined one from F. (H) hiPSC‐CMs heterozygous for mutation R723G were treated with Actinomycin D and fraction of WT per R723G‐mRNA was calculated after 6, 18 and 24 h. (I) Fold change of R723G‐mRNA was calculated for different expected half‐life and compared to the experimentally determined one from H. Statistical significance was calculated by Two‐Way ANOVA, *p < 0.05. **p < 0.01, ***p > 0.001.

We first assessed whether the mutant allele is transcribed with a higher probability. We used an allele‐specific RNA‐FISH analysis for R723G‐ and WT‐mRNA on patient‐derived hiPSC‐CMs. Active transcription of *MYH7* from WT‐and R723G encoding alleles was visualized by an intronic probe set as previously described [3]. Additional probes for either WT‐*MYH7* or R723G‐*MYH7* were designed and co‐hybridized (Figure 1B) using a masked‐oligo approach [5]. Detailed description on methodologies will be made available upon request. Specificity of probes was assessed by hybridization to WT‐hiPSC‐CMs and to RNase‐treated tissue, showing no unspecific binding. Sensitivity was ensured by assessing co‐localization of WT‐ and R723G‐specific probes with intronic probes and including only experiments with an efficacy above 90% (average efficacy: 92.4% ± 3.4% (*n* = 4 hybridizations)). Using this technique, we then analyzed the percentage of nuclei without active transcription sites (aTS), with only WT‐aTS, only R723G‐aTS, and with aTS from both alleles (Figure 1C). Comparable to previous studies where we used one probe set for both alleles [3], we detected 29% ± 9% without aTS. Furthermore, 28% ± 5% of nuclei expressed mRNA only from the WT allele and 23% ± 5% only from the R723G allele. 20% ± 10% of nuclei expressed mRNA from both alleles. Thus, both alleles were transcribed in a comparable fraction of nuclei. On average 46.2% ± 9.4% of actively transcribed alleles encoded for R723G (Figure 1D). Thus, increased fraction of mutant mRNA was not caused by increased transcriptional activity of the R723G‐allele. This finding was supported by an overexpression model in HeLa cells. Transfection of equimolar ratios of expression plasmids encoding either for wildtype (NCBI Accession Number NM_000257.4) or R723G *MYH7*‐cDNA (c.2167C>G) under the CMV‐promoter in a pcDNA3.1 vector (constructs synthesized by GeneArt, Thermo Fisher Scientific) and subsequent allele specific RT‐PCR and allele specific restriction analysis [2] after 24, 48, and 72 h showed no significant deviation from the 50% ratio of R723G‐mRNA over time (Figure 1E).

We next tested whether increased stability of the R723G‐mRNA could underlie the allelic imbalance using a mRNA degradation assay. We again transfected HeLa cells with equimolar ratios of R723G and wildtype expression vectors. After 24 h we stopped transcription by addition of Actinomycin D and harvested mRNA at different time points. Relative fraction of R723G per wildtype mRNA was assessed by RT‐PCR. We detected a statistically significant increase in R723G‐mRNA over time, starting from 50% R723G mRNA rising constantly to 69% at 22 h of transcription inhibition (Figure 1F). This corresponded to a 1.5‐fold increased half‐life of R723G‐mRNA (Figure 1G). To test our hypothesis in a physiological model, we used R723G‐hiPSC‐CMs. Starting from an allelic imbalance of 62% R723G‐ to 38% WT‐mRNA, cardiomyocytes were subjected to Actinomycin D treatment and mRNA was harvested at 6, 18 and 24 h after treatment start. Analysis of allelic mRNA ratios revealed a constant increase in R723G‐mRNA over time to 67% at 24 h of transcription inhibition (Figure 1H). This rise corresponded to a 1.25‐fold increased half‐life of R723G mRNA, which was statistically significant after 24 h (Figure 1I).

Thus, we show that HCM‐mutation R723G increases mRNA stability. Base substitutions can regulate RNA stability by altering access to regulatory RNA‐binding proteins (RBPs) [6]. Mechanisms involve alternative splicing [7] and altered mRNA decay [8]. Either sequence alteration itself or the observed alteration of mRNA structure by mutation R723G [4] presumably alters access to ubiquitously expressed RBPs, which in turn decrease degradation rate. Interestingly, increased RNA stability induced by RNA‐stabilizing proteins has also been associated with hypertrophy development [9]. This finding indicates that altered myosin‐mRNA levels, which are found in hypertrophy development, are at least partly regulated by mRNA stability. Vice versa, increased mRNA stability could contribute to hypertrophy development in HCM, next to the direct effect of the mutation on protein function.

Since increased fractions of mutant protein have been associated with a more malignant disease course, mutant mRNAs have been identified as potential therapeutic targets. One therapeutic approach is therefore to inhibit mutant mRNA expression [10]. Based on our findings, another approach might be the use of modulators of mRNA‐stability as therapeutics. Here, specific RNA‐regions or binding partners have been proposed as therapeutic targets [9]. Thus, future studies have to identify stabilizing interaction partners of R723G‐mRNA and specifically target those.

In summary, our study shows that HCM‐mutation R723G increases mRNA‐stability. This is presumably caused by altered mRNA secondary structure and may therefore provide a novel target for therapeutic interventions to decrease R723G‐mRNA fraction in HCM‐patients.

## Author Contributions

**Kathrin Kowalski:** methodology, investigation, validation, writing – review and editing. **Luisa Schwarze:** investigation, writing – review and editing. **Theresia Kraft:** resources, writing – review and editing. **Judith Montag:** conceptualization, investigation, methodology, supervision, visualization, resources, writing – original draft, funding acquisition.

## Funding

This work was supported by Deutsche Forschungsgemeinschaft, MO‐2238/2‐2.

## Conflicts of Interest

The authors declare no conflicts of interest.

## Data Availability

Data sharing not applicable to this article as no datasets were generated or analyzed during the current study.
